# Stability of Norwalk Virus Capsid Protein Interfaces Evaluated by *in Silico* Nanoindentation

**DOI:** 10.3389/fbioe.2015.00103

**Published:** 2015-07-30

**Authors:** Kevin J. Boyd, Prakhar Bansal, Jun Feng, Eric R. May

**Affiliations:** ^1^Department of Molecular and Cell Biology, University of Connecticut, Storrs, CT, USA; ^2^Department of Chemistry, West Virginia University, Morgantown, WV, USA

**Keywords:** molecular dynamics, *Norovirus*, uncoating, Gō-models, AFM

## Abstract

Norwalk virus causes severe gastroenteritis for which there is currently no specific anti-viral therapy. A stage of the infection process is uncoating of the protein capsid to expose the viral genome and allow for viral replication. A mechanical characterization of the Norwalk virus may provide important information relating to the mechanism of uncoating. The mechanical strength of the Norwalk virus has previously been investigated using atomic force microscopy (AFM) nanoindentation experiments. Those experiments cannot resolve specific molecular interactions, and therefore, we have employed a molecular modeling approach to gain insights into the potential uncoating mechanism of the Norwalk capsid. In this study, we perform simulated nanoindentation using a coarse-grained structure-based model, which provides an estimate of the spring constant in good agreement with the experimentally determined value. We further analyze the fracture mechanisms and determine weak interfaces in the capsid structure, which are potential sites to inhibit uncoating by stabilization of these weak interfaces. We conclude by identifying potential target sites at the junction of a weak protein–protein interface.

## 1 Introduction

Norwalk virus (genus *Norovirus*) is a worldwide human health threat, which causes acute gastroenteritis, resulting in approximately 200,000 annual deaths (Patel et al., 2008). In the US alone, Norwalk virus infects approximately 21 million humans annually, resulting in 800 deaths (Lopman et al., 2012). Treatment of Norwalk virus is primarily limited to rehydration as there are no specific treatments or vaccines available (Iturriza-Gómara and Lopman, 2014). Complicating efforts to develop diagnostic tools to assay potential anti-viral agents is that human noroviruses are not cultivable by standard cell or tissue culture techniques.

Norwalk virus is a small (~38 nm diameter), icosahedral non-enveloped virus with a positive-sense single stranded (ss) RNA genome. The genome encodes for a polyprotein that is post-translationally processed to produce six or seven non-structural proteins (NS1–7) and two structural proteins, VP1 and VP2, the major and minor capsid proteins, respectively. Expression of just the major capsid protein, VP1, will lead to self-assembled virus-like particles (VLPs), which are morphologically and antigenically indistinguishable from native virus (Jiang et al., 1992). A high resolution structure of the Norwalk VLP (NVLP) was determined by X-ray crystallography to 3.4 Å resolution (Prasad et al., 1999).

**Figure 1:**
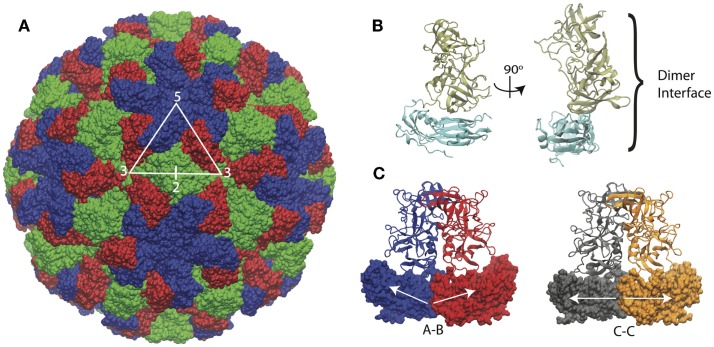
**Norwalk virus capsid structure**. **(A)** The complete capsid is made of 180 subunits, the A subunits (blue) form the pentamers at fivefold symmetry axes. The B (red) and C (green) subunits form the hexamers whose centers are located at the threefold axis. The asymmetric unit is indicated by the triangle, and the twofold axis is labeled as well, which is at the C–C dimer interface. **(B)** The subunit structure is shown with the shell (S) domain in cyan and the protruding (P) domain in tan. **(C)** The A–B and C–C dimers structures are shown. The A–B dimers have an angled interface between the S domains, while the C–C dimers have a flat interface, as indicated by the white arrows.

The structure of the Norwalk capsid has *T* = 3 icosahedral symmetry, consisting of 180 VP1 subunits arranged as 90 dimers. The VP1 protein consists of a shell domain (S) and a protruding domain (P). The S domains form the icosahedral shell, while the P domains (as dimers) form radially extending pillars on the capsid exterior surface. The S domain has a prototypical eight-stranded antiparallel beta-barrel fold, which is commonly seen in plant and animal icosahedral capsids (Abrescia et al., 2012). A flexible linker connects the S and P domains. The capsid assembles from dimers, which adopt different conformations in the capsid. The C–C dimers have a flat conformation, while the A–B dimers adopt a bent interface in the capsid, which is required for the formation of closed shells. The structure of the Norwalk capsid, monomer, and dimers are presented in Figure 1.

Norwalk virus enters cells by endocytosis, through a non-clathrin, non-caveolin mechanism (Perry and Wobus, 2010). After the capsid enters the cell, the genome is released through the (poorly understood) phenomena of uncoating. Given that the capsid has no pores, the uncoating process is presumed to involve a partial or complete disassembly of the capsid to allow for the genome to become exposed in the cytoplasm, where it can undergo translation. An understanding of the mechanical strength of the capsid may lend insights into requirements for disassembly. To this end, single-molecule atomic force microscopy (AFM) nanoindentation experiments have been performed on the NVLPs to gain insights into the capsid mechanical properties. In the AFM experiments, virus particles are adhered to a surface but are kept under solution conditions and then imaged using the AFM probe tip. The height and width of the particles can be measured in the imaging mode. The AFM can then be used in an indentation mode, where force-indentation (FZ) curves can be recorded. From the FZ curves, a spring constant can be obtained from the initial linear region of the curve and the critical force and critical indentation can be determined from the point at which the force drops, indicating a breakage to the capsid (Roos et al., 2010a). A number of viruses have been studied experimentally using AFM nanoindentation including plant viruses (Michel et al., 2006), animal viruses (Uetrecht et al., 2008; Snijder et al., 2013; Li et al., 2014), and bacteriophages (Ivanovska et al., 2004, 2007; Roos et al., 2012). Computational methods have played an important role in interpreting the experimental AFM data and providing theoretical and mechanistic insights into the mechanics and dynamics of viral capsids. These methods range from all-atom molecular dynamics (MD) (Zink and Grubmüller, 2009) to continuum finite element models (FEM) (Klug et al., 2006, 2012). Coarse-grained (CG) molecular models (Arkhipov et al., 2009; Cieplak and Robbins, 2010; Roos et al., 2010b; May and Brooks, 2012; Kononova et al., 2013), which strike a balance between molecular details and computational efficiency, offer the ability to probe longer time scales than traditional MD, but still provide molecular information (unlike FEM) and are particularly well suited for studying capsids.

To date, two AFM studies have been performed on NVLPs, the first of which investigated the influence of pH on the mechanical behavior of the capsid (Cuellar et al., 2010). In that work, it was found that alkaline pH caused a swelling of the capsid and reduced the spring constant by roughly a factor of 5. When the experiments were performed under neutral pH conditions, the measured spring constant was 0.05 N/m. The result differed from a second study, which examined the influence of the protruding domain on the mechanical properties of NVLPs, where the wild-type capsid was found to have a spring constant of 0.30 N/m (Baclayon et al., 2011). One possible explanation of the difference is that in the earlier study (Cuellar et al., 2010), the measured capsid dimensions were inconsistent with the known capsid dimensions, indicating that the particles may have undergone a deformation in the preparation or imaging mode leading to a reduced stiffness. A simulation study utilizing a CG model measured the spring constant of 35 capsids with good agreement for systems with known spring constants (Cieplak and Robbins, 2013). The prediction of the spring constant for NVLP from that modeling study was 0.21 N/m, which is more consistent with the later AFM study of Baclayon et al. (2011).

In this study, we employ a previously developed CG molecular model (May et al., 2011) for the examination of the mechanical nature of NVLPs. Unlike the previous studies on Norwalk virus, we investigate the symmetry/geometrical effects on the capsid mechanics, dissect the molecular interactions, and identify which interfaces in the capsid are most resistant to mechanical stress and which are most responsive. Uncoating inhibitors are viable anti-viral strategies (Liu et al., 2015), and this study may provide new insights into the mechanism and potential target sites for uncoating inhibitors of Norwalk virus.

## 2 Results and Discussion

### 2.1 Mechanical properties

**Figure 2:**
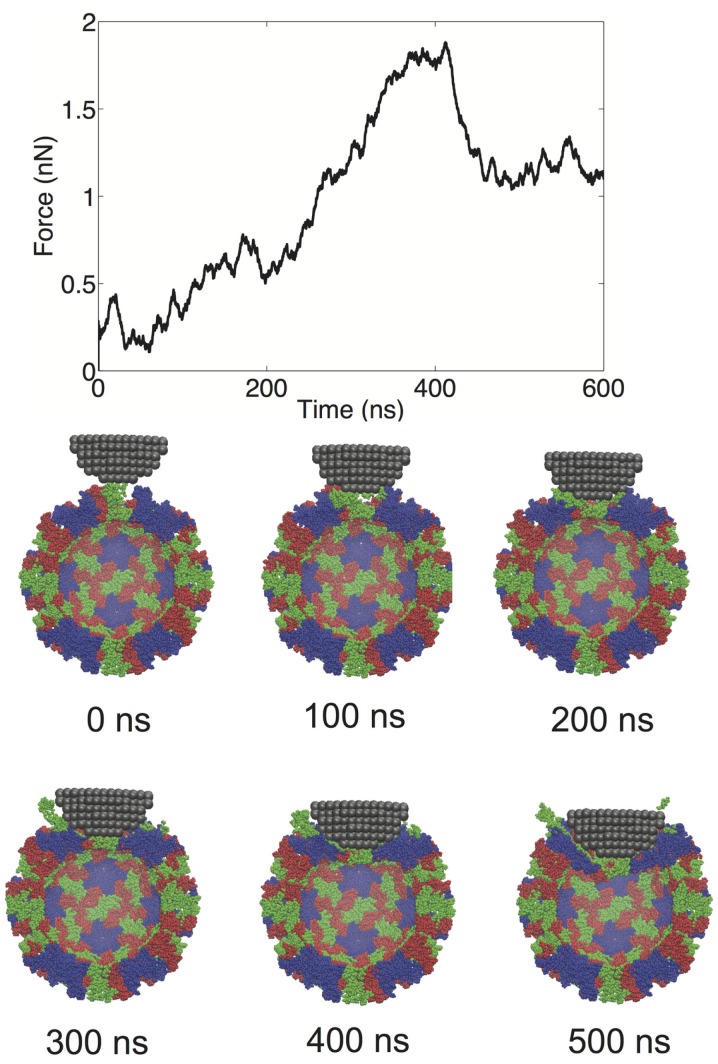
**FT curve and snapshots showing cross sections from a nanoindentation simulation along a twofold symmetry axis**.

We performed a total of nine nanoindentation simulations on a CG model of an NVLP. From these simulations, we could evaluate several parameters related to the material properties of the capsid. These properties include a spring constant obtained from the initial slope of the FZ curve, the maximum force the capsid could withstand (critical force), and the indentation depth at which the critical force occurred (critical indentation). A force vs. time (FT) curve from one of the indentation trials and corresponding snapshots from the trial are presented in Figure 2. The FT curve for the trial in Figure 2 is generated from indentation along a twofold symmetry axis. The resultant curve displays a reasonably linear response region between the initial contact of ~50 ns and the point of critical force of ~400 ns. From the snapshots, distortion to individual protein subunits can be observed, which is due to separation of P from the S domains. The observation that the linker between the P and S domain is highly flexible is supported by antibody binding studies in which the P domain was shown to be “lifted” away from the S domain upon antibody binding (Katpally et al., 2008, 2010).

**Figure 3:**
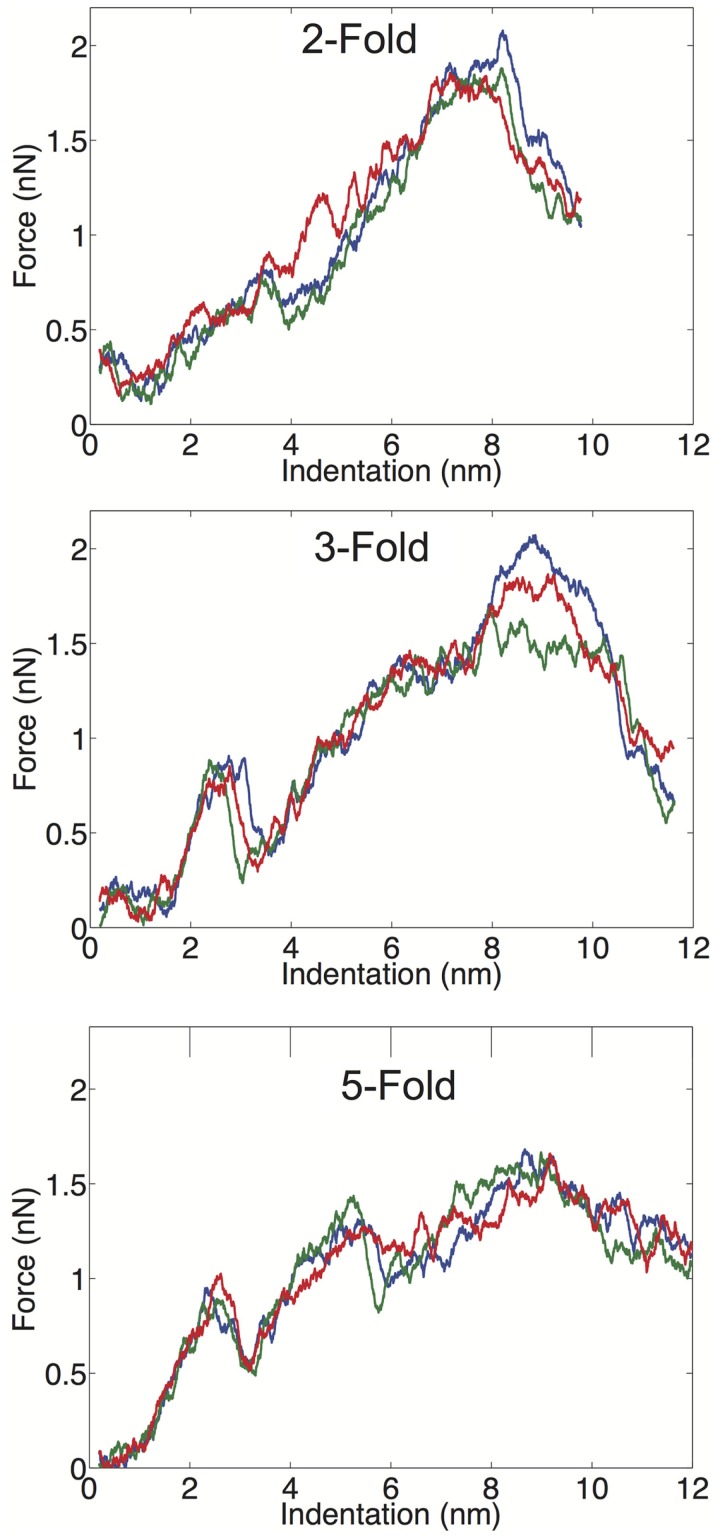
**FZ curves along the twofold (top), threefold (middle), and fivefold (bottom) symmetry axes**.

We examined the geometrical variation in mechanical properties by performing nanoindentation simulations along a twofold, threefold, and fivefold symmetry axis; each orientation was indented three times in independent simulations. We observed significant consistency between trials along a given symmetry axis, but significant differences between the three different orientations. Each of the FZ curves from the three different orientations is presented in Figure 3. The threefold and fivefold axes simulations display a significantly more non-linear character than the twofold axis simulations, each showing a more pronounced local maximum around 2.5 nm of indentation. The fivefold axis displays a second local maximum around 5 nm, and requires considerably less maximum force than the other symmetry axis simulations. In the experimental study by Cuellar et al. (2010), they discuss three different characteristic FZ curve shapes: linear (65%), multiple slopes (17%), and sudden force drops (18%). It is possible their observation of non-linear FZ curves was due to orientational differences, which would be undetectable by AFM. In the later study by Baclayon et al. (2011), they do not discuss non-linearities in their FZ curves, but they do report a broad range of spring constants ranging from 0.1 to 0.65 N/m, indicating that there was non-uniformity in the stress response.

We have computed the average material properties from the nine indentation trials and these properties compare favorably to the results reported by Baclayon et al. (2011). When we compute average properties, we consider the probability of indenting along a given axis, since there are unequal numbers of twofold, threefold, and fivefold axes in an icosahedral shell. There are 30 twofold, 20 threefold, and 12 fivefold sites on an icosahedron, and we use these probabilities in our averaging (see Materials and Methods). Our average spring constant is *k* = 0.21 ± 0.05 N/m, which is in good agreement with the experimentally reported value of 0.30 ± 0.09 N/m, the errors are the SDs of the distribution. The SD of the simulation data is based upon duplicating the measured values such that the ratio of values in the distribution is consistent with the symmetry probabilities. The range of spring constants, we measured was between 0.12 and 0.26 N/m, while the experimental range was between 0.10 and 0.65 N/m. While we do not observe spring constants as stiff as seen experimentally, the high stiffness measurements are low probability events, and we may not have a large enough sample size to observe these large deviations from the mean value. Overall, the average, *k* and the width of the distribution are well captured in the CG model and our mean *k* value is in exact agreement with the previous simulated measurement using a similar (but not identical) CG model (Cieplak and Robbins, 2013).

The average critical force in our simulations is *f_c_* = 1.6 ± 0.2 nN, and the critical indentation value is 6.8 ± 0.7 Å. Both of these metrics are overestimated in our simulations; the experimentally determined values are 1.1 ± 0.9 nN and 4.1 ± 3.7 Å, for the critical force and critical indentation values, respectively. Again the errors are the SD of the distributions, and it can be observed that the simulated and experimental distributions are overlapping. The increased mean value for the critical force and indentation in our simulations is likely due to the high force loading rate that we have applied. The experimental loading rate is 50 nm/s, which is not feasibly reproduced in MD simulations (even with a CG model). MD nanoindentation simulations on virus capsids have been performed in the range of 10^4^ nm/s (Kononova et al., 2013) to 10^10^ nm/s (Zink and Grubmüller, 2009); our simulations are performed at a loading rate of 10^7^ nm/s. It should be noted that the rate in our simulations (and other CG simulations) is not a true rate, because the dynamics are accelerated in the CG model due to a smoothed energy surface. While it is difficult to interpret the meaning of the time-step in Gō-like models, it is likely that the effective dynamics are at least three orders of magnitude faster than the actual simulation time (Buck and Bystroff, 2011), which would put our loading rate in the 10^4^ nm/s range. A previous study using a CG model (though not a Gō-model) showed that varying the loading rate from 10^5^ to 10^7^ did not have drastic effects on the slopes of the FZ curves (Arkhipov et al., 2009). While a slower loading rate in our simulations may improve the agreement in the calculated mechanical properties and reduce some of the non-linear effects seen in the FZ curves, overall the model performs well and is adequate for addressing the relative strength of the protein–protein interfaces in the capsid, which is the main focus of this study.

### 2.2 Interface stability analysis

**Figure 4:**
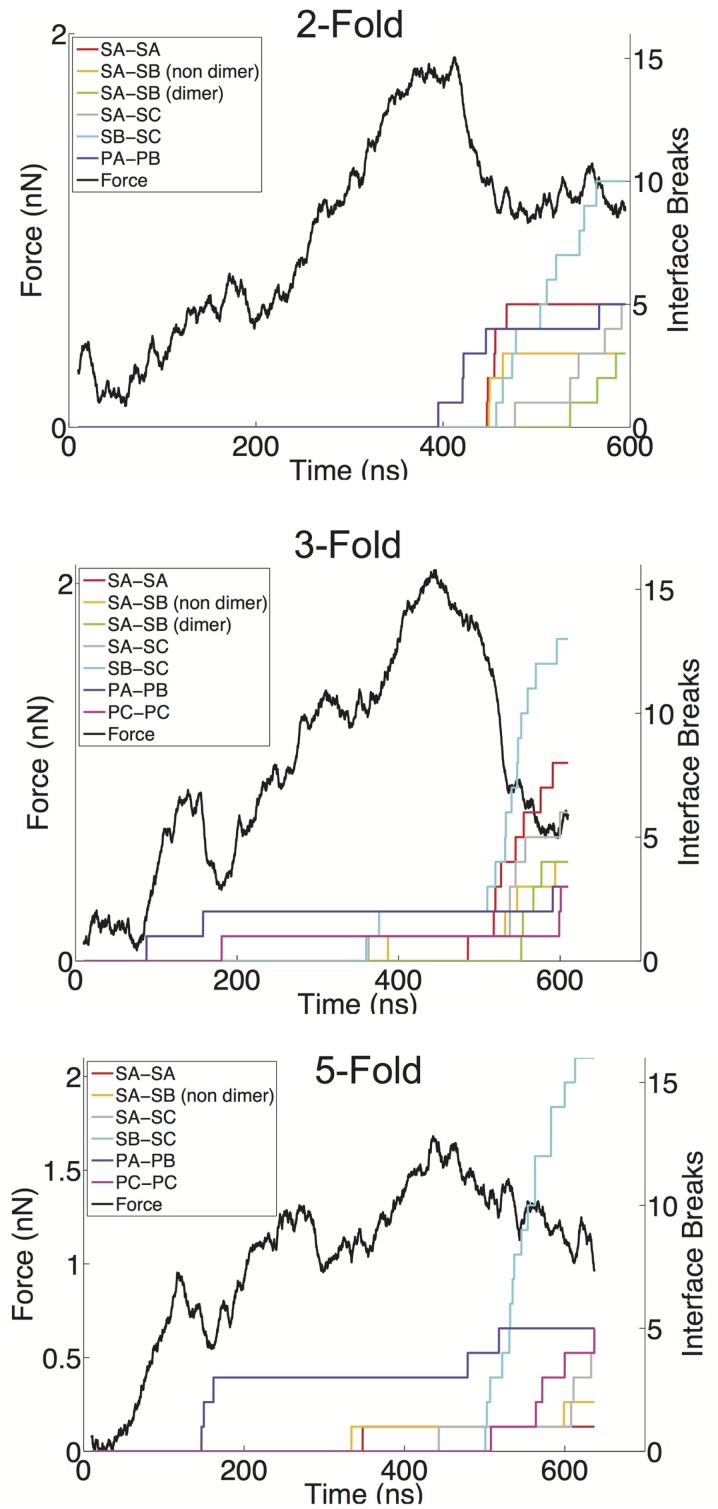
**FT curves overlaid with the interface breaks during the simulation**. A single trial of indentation along the twofold (top), threefold (middle), and fivefold (bottom) symmetry axes is presented. All interfaces, which accumulated at least two breaks, are shown. The colored lines represent the cumulative sum of the number of interfaces, which become broken, for various interface types. Interfaces are considered broken once the fraction of native contacts in that interface has dropped below 50% for a consecutive period of 5 ns.

We have defined a metric for monitoring the breakage of protein–protein interfaces (see Materials and Methods), to provide an understanding of the relative strength of various interfaces in the Norwalk virus capsid. In brief, the approach monitors the contacts between the specific protein–protein interfaces in the capsid. When 50% of the contacts that are present in the x-ray structure (native contacts) in a given interface have been become substantially separated (≥1.5× the native contact distance), we consider that interface to be broken. Through the course of the simulation, we can tabulate the number of various interfaces, which have become disrupted. We have overlaid the FT data with the interface breaks as shown in Figure 4 and in Figure S1 in Supplementary Material. The interfaces are categorized as either shell domain (S) or protruding domain (P) and are enumerated between the A, B, and C subunits. In the case of the shell domain interactions between the A and B subunit (SA–SB), we distinguish between the dimer-forming interface and the non-dimer interface. We can draw several inferences from these analyses. In eight of the nine simulations, the interface with the most breaks at the end of the simulation is the SB–SC interface. The SB–SC interfaces are what make up the hexamers, but they do not contain P domain interfaces. Capsid assembly is believed to proceed by nucleation followed by addition of A–B and C–C dimers, implying that dimers are the strongest interface (Prasad et al., 1999). Therefore, the observation that a non-dimer forming interface (SB–SC) would be comparatively weak and exhibit a large number of breaks is very reasonable. Furthermore, using native ion mass spectrometry, it was shown that a pentamer of dimers (type A–B) is the likely assembly nucleus (Uetrecht et al., 2010). This implies that pentamers are stronger than hexamers and therefore supports our prediction of weak SB–SC interfaces, and hence weak hexamers (relative to pentamers).

Another observation from our indentation simulations is the robustness of the C–C interfaces, particularly the SC–SC interaction. The C–C dimer lies at a twofold axis and spans two neighboring hexamers. In only one simulation is an SC–SC interface broken (and only one actual interface is broken), compared to six simulations showing SA–SB dimer interfaces breaks, where as many as five such interfaces break in a given simulation. The dimer protruding domains show a similar characteristic in that in only one of the simulations are there more PC–PC breaks than PA–PB breaks, and the total amount of PA–PB breaks summed over the nine simulations is considerably greater than the total number or PC–PC breaks. A simple explanation for the C–C dimer being more stable is that the flat interface in the C–C configuration is consistent with the solution configuration of the dimer, and hence the lowest free energy configuration. The A–B dimer must be bent to introduce curvature to the capsid and therefore is a higher energy (less stable) state. It has been speculated that the VP2 protein acts to stabilize the bent A–B state (Vongpunsawad et al., 2013), further supporting the A–B configuration as a perturbed state away from the native state.

The breakage data also provide insights into the non-linear regions in the force response curves. As can be seen in the threefold and fivefold data in Figure 4, the local force peak and break around 200 ns can be attributed to protruding domain breaks. In the threefold case, we can see both PA–PB and PC–PC breakages occurring, whereas in the fivefold, only PA–PB breakages are occurring in the initial stages. It is interesting that in the threefold and fivefold simulations, the indentation is centered in a “valley” (either the center of a pentamer or hexamer) surrounded by a protruding domains. Whereas in the twofold simulations, the indentation is centered over a PC–PC interface, yet no early-stage protruding domain breaks are being observed. This observation may indicate that protruding domains are more susceptible to a lateral or shear deformations as compared to a direct compression. It also appears that the capsid behaves as a uniform shell when being indented over the twofold axis, where the capsid undergoes a cooperative (catastrophic) failure (buckling) (Lidmar et al., 2003; Klug et al., 2012) when the critical force is reached. Whereas along the threefold and fivefold axes, the capsid behaves more discretely allowing for minor failures to occur before the critical force is reached.

### 2.3 Putative target sites for B–C interface stabilization

**Figure 5:**
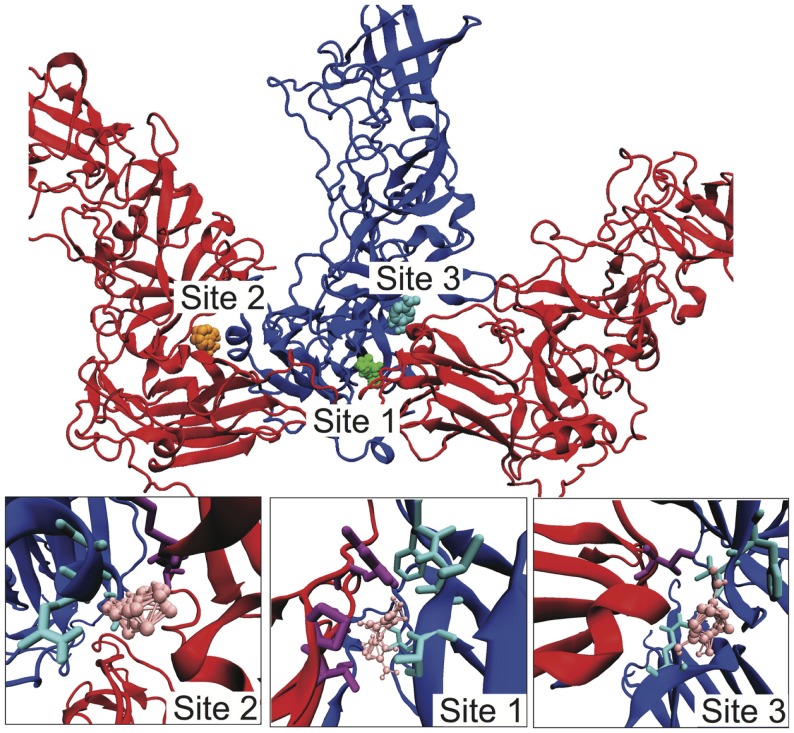
**Identification of potential SB–SC stabilization sites**. Using FTMap three binding sites were identified, which bound to residues in both subunits B and C. Subunit B is drawn in blue and subunit C is red. In the top image, the probes at site 1 are green, at site 2 orange, and site 3 cyan. In the lower zoom-in images, the organic probes are pink, residues with an atom within 3 Å of a probe is drawn in as cyan for subunit B, and purple for subunit C.

Given our identification of the SB–SC interfaces as weak and a potential site for stabilization to act as an uncoating inhibitor, we examined the SB–SC surface to identify potential drug target sites. We identified druggable “hot spots” using the FTMap server, which uses organic probe molecules to identify consensus binding sites on a protein surface (Brenke et al., 2009). FTMap identified 16 druggable sites over the entirety of the B–C subunit proteins, but only three of those sites spanned the SB–SC interface. Those three sites are shown in Figure 5, including zoomed-in images of the binding sites, depicting the interacting residues. Table 1 lists the residues having an atom with 3 Å of an organic probe in each of the binding sites.

**Table 1:** **Target residues^a^ in the three putative binding sites to stabilize the B–C interface**.

Binding site	Subunit B residues	Subunit C residues
1	ALA114, PHE200, VAL201, VAL202, ALA203	GLY121, THR188, PRO189, ARG191
2	ALA140, GLN141, LEU144	VAL61, GLN62
3	GLN62, PHE503, VAL504	THR130

*^a^Target residues are those residues with an atom within 3 Å of a probe molecule*.

## 3 Conclusion

We have performed nanoindentation simulations using a structure-based CG model of the Norwalk virus capsid. This is a computationally efficient model, which can capture much of the important physics of a virus capsid, as shown by the agreement between these computations and measured mechanical properties of the capsid. These simulations shed light on details, which cannot be observed by experimental methods, including causes of non-linearities and the ability to resolve which interfaces are being disrupted. Our findings regarding the relative stability of regions of the capsid are well supported by previous experimental studies including mass spectrometry analysis of assembly intermediates (Uetrecht et al., 2010). Based upon the identification of a weak interface (SB–SC), we performed computational solvent mapping and identified potential hot spots, which demonstrate the plausibility that small molecules could bind at this protein–protein interface.

## 4 Materials and Methods

The model for the NVLP is based upon the “flavored” Gō-Model, introduced by Karanicolas and Brooks (2002, 2003). The model was previously modified to study virus capsids dynamics (May et al., 2011), and the same model is implemented in this study. Further details can be found in the previous study, but we will reiterate the key features of the model here. A potential is built such that a known structure is at the global energy minimum. For our study, the known structure is the x-ray structure of the Norwalk virus capsid pdbid:1ihm (Prasad et al., 1999). The Gō-model is built by identifying native contacts from the all-atom structure and then mapping those interactions onto a Cα-based model. The contact potential has the form:

(1) $$V_{\mathrm{ij}}=\varepsilon_{\mathrm{ij}}\;\left[5{\left(\frac{\sigma_{\mathrm{ij}}}{r_{\mathrm{ij}}}\right)}^{12}-6{\left(\frac{\sigma_{\mathrm{ij}}}{r_{\mathrm{ij}}}\right)}^{10}\right]$$

where the *r_ij_* is the separation between Cαs of residue *i* and *j*, σ is the Cα separation distance in the initial structure (the native contact distance), and ϵ*_ij_* is the strength of the pair interaction, which is based upon the Miyazawa–Jernigan knowledge-based potential (Miyazawa and Jernigan, 1996). Any non-native interactions are purely repulsive $\left(r_{\mathrm{ij}}^{-12}\right)$, and pseudo bond, angle, and dihedral terms are included in accordance with the original model.

The simulations are conducted in the GROMACS simulation package version 4.5.5 (Van Der Spoel et al., 2005) using a Langevin integrator with 1 ps^–1^ friction factor, 10 fs time step and maintained at 270 K. Non-bonded forces are computed for pairs separated by up 33 Å, and the data were saved at 100 ps intervals. The total size of the capsid model was 89,700 CG particles. The AFM probe tip was modeled as a hemisphere with a radius of ~9 nm containing 550 identical particles with masses of 2 kDa arranged in a cubic lattice with a distance of 1.5 nm between particles. Adjacent particles in the lattice were bonded to each other, and movement is frozen in the *x* and *y* direction. The interaction between the virus and tip follows the 12–10 potential, where the interaction becomes repulsive at separations below 15 Å. The tip is moved by applying an umbrella potential with a spring constant of 200 kJ/(mol*Å^2^) at a rate of 0.00002 nm/ps. The force acting on the virus is determined from the difference between the location of the umbrella potential and the center of mass of the tip, as shown in equation 2,

(2) $$F\left(t\right)=k\left(z\left(t\right)-z_{0}-v\times t\right)$$

where *z*_0_ is the initial tip center of mass position and *v* is the umbrella velocity. In the force curves presented, the force data are smoothed by averaging over 100 data points (10 ns). The spring constants were determined from a linear least squares fitting of the FZ curves between the region starting at 75 ns and up to the location of maximum force, using MATLAB. The critical force and critical indentation were the calculated as the change in force or indentation between the point of maximum force and the respective values at 75 ns. Discarding of the first 75 ns was done because the tip is not firmly contacting the virus before that point. During the indentation, beads in the bottom half of the viruses are fixed at their initial positions, to mimic the effect of adherence to a substrate, similar to other work (Kononova et al., 2013). Geometrical averaging is performed by considering the ratio of twofold, threefold, and fivefold axis in the capsid. We compute averages for each of the orientations (e.g., *k*_2-_*_fold_*, *k*_3-_*_fold_*, *k*_5-_*_fold_*) and then compute the geometrical average by

(3) $$k_{\mathrm{avg}}=\frac{1}{31}\left(15k_{2-\mathrm{fold}}+10k_{3-\mathrm{fold}}+6k_{5-\mathrm{fold}}\right).$$

To estimate the SD of the distribution, a distribution is generated, which has the measured twofold, threefold, and fivefold values repeated in a ratio consistent with the geometry.

The interface breaks were determined using in-house software utilizing the Pteros 2.0 library for C++ (Yesylevskyy, 2012). The native contacts for each interface were culled from the forcefield and a contact was considered to be formed if it was within 1.5 times the native contact distance. If 50% of the native contacts in a given interface were broken for a consecutive 5 ns period, the interface was considered broken. The druggable hotspots were determined by submitting a pdb containing neighboring B–C subunits to the FTMap server (ftmap.bu.edu) (Kozakov et al., 2015).

## Author Contributions

EM built the model, designed the simulations, and wrote the manuscript. JF aided in designing and implementing the coarse-grained model. KB implemented and conducted the nanoindentation simulations. PB designed and performed analyses to monitor the integrity of interfaces during the simulations.

## Conflict of Interest Statement

The authors declare that the research was conducted in the absence of any commercial or financial relationships that could be construed as a potential conflict of interest.
