# Supplementary material for: Stability of Norwalk Virus Capsid Protein Interfaces Evaluated by in Silico Nanoindentation
Source: Front Bioeng Biotechnol. 2015 Jul 30;3:103. doi: 10.3389/fbioe.2015.00103 (PMC4520240; doi:10.3389/fbioe.2015.00103)
Supplement: Supplementary file 1 [file image_1.pdf]

Supplementary Material

Stability of Norwalk virus capsid protein interfaces evaluated by *in-silico* nanoindentation

Kevin J. Boyd[1], Prakhar Bansal[1], Jun Feng[2], Eric R. May[1]

[1]Department of Molecular and Cell Biology, University of Connecticut, Storrs, CT, USA
[2]Department of Chemistry, West Virginia University, Morgantown, WV, USA

*** Correspondence:** Eric R. May, Department of Molecular and Cell Biology, University of Connecticut, Storrs, CT, USA. eric.may@uconn.edu

1. Supplementary Figures and Tables

A supplementary figure is included which contains the overlay of the force and breakage data for the trials not presented in Fig. 4 of the main text.

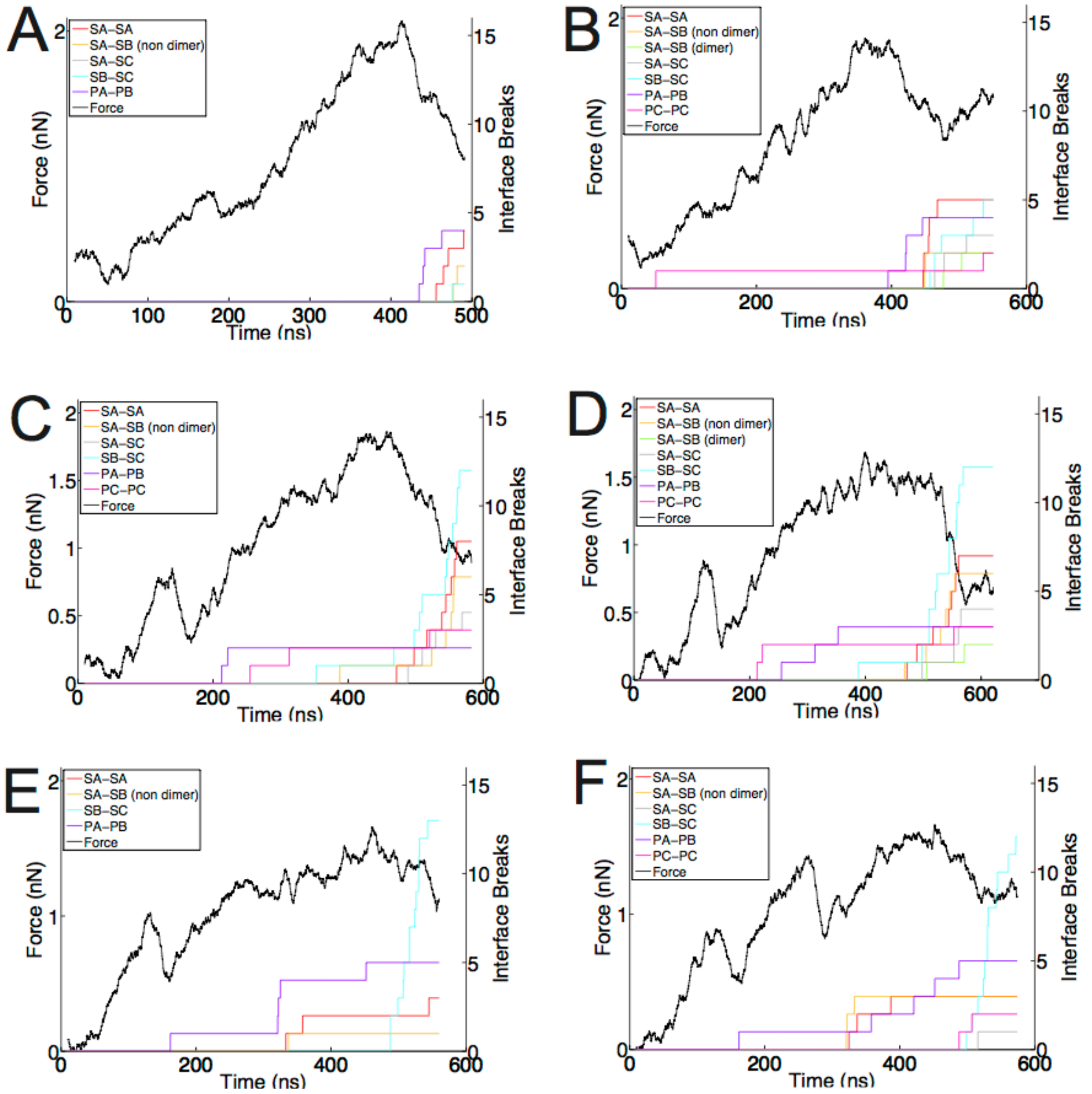

Supplementary Figure 1. Additional FT curves overlaid with interface breaks data. A-B are two-fold trials, C-D and three-fold trials and E-F are five-fold trials.

.
